# Pramipexole use and the risk of pneumonia

**DOI:** 10.1186/1471-2377-12-113

**Published:** 2012-09-29

**Authors:** Pierre Ernst, Christel Renoux, Sophie Dell'Aniello, Samy Suissa

**Affiliations:** 1 Center for Clinical Epidemiology, Lady Davis Research Institute - Jewish General Hospital, 3755 Cote Ste-Catherine, Montreal, QC, H3T 1E2, Canada; 2 Division of Pulmonary Medicine, Jewish General Hospital, Montreal, QC, Canada; 3 Departments of Epidemiology and Biostatistics, and of Medicine, McGill University, Montreal, QC, Canada

**Keywords:** Anti-parkinsonian drugs, Drug safety, Parkinson's disease, Restless leg syndrome, Observational study

## Abstract

**Background:**

Patients with Parkinson's disease have an elevated risk of pneumonia and randomized trials suggest that this risk may be increased with the dopamine agonist pramipexole. It is uncertain whether pramipexole or other dopamine agonists increase the risk of pneumonia.

**Methods:**

We used the United Kingdom's General Practice Research Database (GPRD) to identify users of anti-parkinsonian drugs, 40–89 years of age, between 1997 and 2009. Using a nested case–control approach, all incident cases hospitalised for pneumonia were matched with up to ten controls selected among the cohort members. Rate ratios (RR) and 95% confidence intervals (CI) of pneumonia associated with current use of dopamine agonists were estimated using conditional logistic regression, adjusted for covariates.

**Results:**

The cohort included 13,183 users of anti-parkinsonian drugs, with 1,835 newly diagnosed with pneumonia during follow-up (rate 40.9 per 1,000 per year). The rate of pneumonia was not increased with the current use of pramipexole (RR 0.76; 95% CI: 0.57-1.02), compared with no use. The use of pramipexole was not associated with an increased rate of pneumonia when compared with all other dopamine agonists collectively (RR 0.85; 95% CI: 0.62-1.17).

**Conclusions:**

The use of pramipexole does not appear to increase the risk of pneumonia.

## Background

Parkinson's disease generally affects the elderly with a prevalence of around 2% among the northwestern European population over 65 years of age [1,2]. Dopamine agonists have become first-line agents for the symptomatic treatment of Parkinson's disease, but are also used in other conditions such as restless legs syndrome. Parkinson's disease has been associated with significant increases in deaths from pneumonia and aspiration pneumonia, possibly resulting from the combination of chronic immobilization and swallowing impairment [3-6].

Adverse events of pneumonia have been noted in association with pramipexole use in various trials conducted by Boehringer-Ingelheim. The signal of a potential risk of pneumonia arose in pooled data from 21 placebo controlled randomized trials of pramipexole conducted in Parkinson's disease and restless legs syndrome [7]. The pooled analysis, involving 3,662 patients on pramipexole and 2,469 on placebo, observed a numerically increased rate of adverse events of pneumonia (10.7 versus 3.6 per 1000 patient-years; rate ratio 2.5; 95%CI: 0.9- 7.0). The adverse event data on pneumonia were, however, limited by the small number of events in the clinical trials and the fact that pneumonia was only one of several adverse events reported in these trials, so that an association could have been the result of chance. Moreover, no associations between treatment for Parkinson's disease and pneumonia have been reported in the literature. Nevertheless, the possibility of an association between pramipexole and pneumonia remains and also raises the question of an increase in the risk of pneumonia with other dopamine agonists.

We therefore conducted a population-based cohort study to assess whether the use of pramipexole and of other dopamine agonists increases the risk of pneumonia.

## Methods

We used a population-based cohort study design with a nested case–control analysis. This approach was necessary to account for the time-varying nature of anti-parkinsonian drug exposure.

### Data source

Data were obtained from the United Kingdom's General Practice Research Database (GPRD), which includes computerized medical records of more than 10 million patients from more than 500 general practices in the United Kingdom. General practitioners, using standardized recording of medical information, record data on the patient's demographic characteristics, symptoms, history, medical diagnoses, and drug prescriptions, as well as details of referrals to specialists and hospitals. The completeness and validity of the recorded information on diagnoses and drug exposures, as checked on an ongoing basis by staff of the GPRD, have been shown in several studies [8-10].

Recently, the GPRD gained approval to enable record linkage of GPRD data with other healthcare databases via the patient's NHS (National Health Service) number, sex, date of birth and Post Code. Specifically, the Hospital Episode Statistics (HES) database records information on all hospitalisations, including data on length of stay, ward types, as well as extensive disease and procedure coding. The linkage between the GPRD and the HES databases applies to approximately half of the practices contributing to the GPRD. The GPRD is the most validated of all databases used for drug safety and research on the study of numerous diseases, including Parkinson's disease [11], and community-acquired pneumonia [12,13].

### Study population

**Figure 1:**
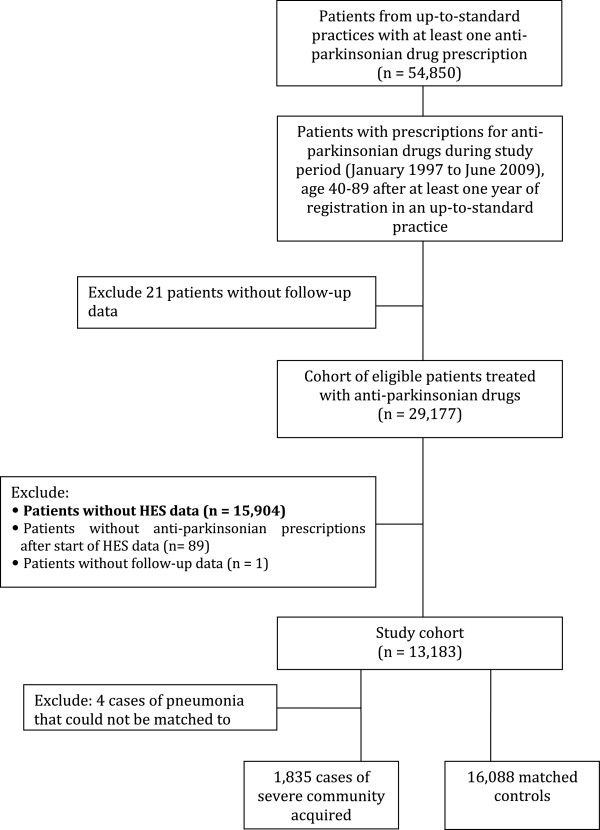
**Flowchart describing the selectionof the cohort of13,183 users of anti-Parkinsoniandrugs, 40–89 years ofage, observed between January1, 1997 and June30, 2009, identified fromthe United Kingdom's GeneralPractice Research Database (GPRD).**

The study base population included all users of anti-parkinsonian drugs, registered with an up-to-standard GPRD practice and who were 40 to 89 years of age between January 1, 1997 and June 30, 2009 (Figure 1). This study period was selected to encompass the date pramipexole (Mirapexin) was approved (February 1998) and subsequently entered the UK market. The drugs examined are the dopamine precursor levodopa, the monoamine oxidase inhibitors selegiline and rasagiline, and the dopamine agonists bromocriptine, cabergoline, lisuride, pergolide, pramipexole, ropinirole, and rotigotine. It is noteworthy to mention that these medications are not only used for treatment of Parkinson's disease, but also given for the Parkinsonian syndrome, restless legs syndrome, hyperprolactinemia and acromegaly.

Cohort entry was defined by the first prescription of one of these drugs after the latest date among January 1, 1997, the date of the patient's 40th birthday, and one year after the date of the patient's registration with the practice, all after the up-to-standard date of the practice. Patients who received any of these drugs in the year before cohort entry were labeled as prevalent users, while the others were considered new users. The follow-up time of the cohort members ended at the time of the occurrence of the first of the following events: a diagnosis of pneumonia, the 90th birthday, death, the end of the patient's registration with the practice or of the contribution of data by the general practice, or the end of the study period (June 30, 2009). Since the definition of the pneumonia outcome required information from hospitalizations, we excluded all subjects whose practice was not linked to the HES database of hospitalization records.

### Cases (endpoint)

Cases of pneumonia were defined by a first clinical diagnosis of severe community acquired pneumonia during cohort follow-up identified by a hospitalization for pneumonia or acute lower respiratory infection identified using ICD-10 codes. A record of a pneumonia or acute lower respiratory infection diagnosis without hospitalization was not included in the case definition. Patients were not included as cases if pneumonia developed during hospitalization (i.e., hospital-acquired pneumonia), so that only diagnoses of pneumonia or acute lower respiratory infection on the day of admission or the following day were considered as eligible. The date of the first recorded diagnosis was defined as the index date.

### Controls

Because of the time-varying nature of drug exposure, we used a nested case–control approach to data analysis. For each case, up to 10 controls per case were randomly selected among the cohort members from the patients at risk of developing pneumonia at the index date of the case. Controls within the risk set were matched to the case on the diagnosis (Parkinson's disease, Parkinsonian syndrome, restless legs syndrome, hyperprolactinemia and acromegaly), age at index date (±5 years), sex, prevalent or new user status at cohort entry, and year of cohort entry. For 32 cases for which no controls could be found, the matching criteria were widened for year of cohort entry (±1 year) and age (±10 years), leaving four cases that were excluded because no match could be found. The controls were assigned the index date of the case they were matched to.

### Exposure

All prescriptions for the dopamine agonists under study were identified. These included the non-ergot derived pramipexole, ropinirole, and rotigotine, and the ergot-derived bromocriptine, cabergoline, lisuride, and pergolide. For all cases and controls, current exposure to a dopamine agonist was defined as a prescription ending after, or within 30 days prior to, the index date.

### Covariates

To control for potential confounding, we identified several factors in addition to the matching factors that include the drug indication, age, sex, new user status and year of cohort entry. Thus, we also obtained data on use of alcohol, smoking status, body mass index (BMI), as well as co-morbidities associated with pneumonia, all prior to cohort entry. In particular, these include chronic obstructive pulmonary disease (COPD) including chronic bronchitis, asthma, diabetes, cerebrovascular disease, coronary heart disease, heart failure, rhythm irregularity, cardiac valve condition, lung cancer, other cancer, depression, motor neuron disease, bipolar disorder, psychosis, dementia, epilepsy, hypertension, renal failure, anemia, peripheral edema, pneumonia hospitalized or not, all occurring any time prior to cohort entry. In addition, other drugs used in this context including the dopamine precursor levodopa, the monoamine oxidase inhibitors selegiline and rasagiline, anticholinergic drugs, the catechol-O-methyl transferase (COMT) inhibitors and amantadine, were considered concurrently to the drugs under study. The use of oral and inhaled corticosteroids, other respiratory medications, pneumococcal and influenza vaccination, ACE-inhibitors, ARBs, diuretics, NSAIDS, PPIs, barbiturates, anxiolytics, antipsychotics, antidepressants, opiates, mood stabilizers, immunosuppressants associated with an increased risk for pneumonia, was identified in the year prior to index date. We adjusted for antibiotics used in the year prior to the index date, but excluded any prescriptions given in the 2 week before the index date as it may be intended for early symptoms of pneumonia among the cases. Finally, we also considered duration of disease, calculated as the time between the date of the first anti-parkinsonian drug and cohort entry.

### Data analysis

The overall rate of pneumonia was computed for the cohort using the person-time of follow-up. Because of the time-varying nature of anti-parkinsonian drug use, the nested case–control approach to analysis was used to estimate the incidence rate ratios of pneumonia from odds ratios calculated by conditional logistic regression, both crude and adjusted for the potential confounders. For the primary analysis, the incidence rate ratio was estimated for current use of pramipexole compared with the reference category of current use of other dopamine agonists. For the secondary analysis, the effect of current use of each dopamine agonist was estimated using a single regression model where current use of each drug, compared with non-current use of the drug, was included as an independent factor. Missing confounder data for smoking and BMI (15.6% and 26.2% respectively in controls) was considered by creating a separate category for the missing data.

Several sensitivity analyses were performed. First, we restricted the analysis to the cohort of subjects for whom the indication for treatment was Parkinson's disease and to the sub-cohort with at least two prescriptions for an anti-parkinsonian drug. Second, we varied the 30-day definition of current use of the drugs by using exposure time windows of 0, indicating prescriptions ending at or after the index date, as well as 14, 60 and 90 days. Third, to examine the possibility of channeling bias where the choice of medication might be influenced by a history of pneumonia or prior use of anti-parkinsonian medication, we carried out analyses stratified by history of pneumonia prior to cohort entry and by the type of anti-parkinsonian medication received prior to the 180-day period before the index date. Finally, we performed an analysis using a more restricted definition of pneumonia, that is, eliminating cases of pneumonia due to aspiration of liquids or solids (ICD-10: J69), lower respiratory infection without mention of pneumonia, and either pneumonia or lower respiratory infection with a concurrent diagnosis of heart failure.

The study protocol was approved by the Independent Scientific Advisory Committee (ISAC) for the U.K. Medicines and Healthcare Products Regulatory Agency and the Ethics Committee of the Jewish General Hospital. All data used in this study was anonymized.

## Results

The cohort included 13,183 patients treated with anti-parkinsonian drugs, after excluding 15,904 subjects whose practice was not linked to the HES database of hospitalization records. At cohort entry, patients were 71.7 (±12.0) years of age and 49.4% were men, while 65.1% were newly treated with anti-parkinsonian drug. The mean duration of cohort follow-up was 3.4 (±2.9) years during which 1,835 patients were diagnosed with pneumonia, for an overall incidence rate of pneumonia of 40.9 per 1,000 per year. Among the 1,835 cases of pneumonia, pneumonia was the principal diagnosis in 45%, while 31% had a diagnosis of acute lower respiratory infection, 13% a diagnosis of pneumonitis due to aspiration of liquids and solids, and for the remaining 11%, the pneumonias or acute lower respiratory infection appeared concurrently with a diagnosis of heart failure.

Table 1 describes the characteristics of these cases of pneumonia and their matched controls. The cases were diagnosed with pneumonia at 79 years of age, 62% were male, 57% were newly treated with an anti-Parkinsonian drug, and over 60% had Parkinson's disease as the indication, with controls matched on these characteristics. For 27%, the indication was not mentioned. As expected, the pneumonia cases had greater co-morbidity prior to cohort entry.

**Table 1:** **Comparison of cases ofpneumonia and their matchedcontrols**

	**Cases**	Controls*
Number	1,835	16,088
Age at index date (years; mean ± sd)	79.2 ± 8.0	78.6 ± 7.8
Male sex (%)	1,137 (62.0)	10,000 (62.0)
New-user at cohort entry (%)	1,053 (57.4)	9,251 (57.4)
Disease duration at cohort entry among prevalent users (yrs; mean ± sd)	3.1 ± 2.9	3.1 ± 2.9
**Indication for treatment** (%)		
Parkinson's disease	1,114 (60.7)	10,758 (60.7)
Parkinsonian syndrome	127 (6.9)	649 (6.9)
Restless leg syndrome	85 (4.6)	644 (4.6)
Hyperprolactinemia/acromegaly	21 (1.1)	73 (1.1)
Not mentioned	488 (26.6)	3,964 (26.6)
Body mass index ≥ 30 (%)†	211 (16.4)	1,624 (14.0)
Ever smoking (%)†	685 (45.7)	5,300 (39.7)
Alcohol abuse (%)	104 (5.7)	701 (4.4)
**Co-morbidity prior to cohortentry (%)**		
Cerebrovascular disease	339 (18.5)	2,109 (13.0)
Ischemic heart disease	419 (22.8)	3,462 (21.1)
Hypertension	622 (33.9)	5408 (33.4)
Heart Failure	175 (9.5)	880 (5.2)
Diabetes	172 (9.4)	1031 (6.5)
Rhythm irregularity	224 (12.2)	1652 (10.1)
Cardiac valve condition	22 (1.2)	162 (1.0)
Peripheral edema	428 (23.3)	3109 (19.0)
Chronic obstructive pulmonary disease	315 (17.2)	2023 (12.4)
Asthma	205 (11.2)	1447 (9.0)
History of prior Pneumonia	127 (6.9)	564 (3.6)
Lung Cancer	4 (0.2)	25 (0.1)
Other Cancer	305 (16.6)	2251 (14.0)
Depression	265 (14.4)	2071 (12.7)
Anemia	208 (11.3)	1483 (9.4)
Renal Failure	84 (4.6)	473 (2.8)
Dementia	153 (8.3)	918 (5.5)
Motor Neuron Disease	1 (0.1)	11 (0.1)
**Medication use in yearprior to index date(%)**		
Diuretics	828 (45.1)	5911 (36.6)
ACE- inhibitors	383 (20.9)	3084 (18.9)
Angiotensin receptor blocker	96 (5.2)	845 (5.2)
Proton-pump inhibitors	563 (30.7)	3566 (22.6)
NSAIDS	357 (19.5)	3093 (19.5)
Oral corticosteroids	247 (13.5)	962 (6.2)
Inhaled corticosteroids	243 (13.2)	1331 (8.4)
Other respiratory medications	372 (20.3)	1844 (11.8)
Antibiotics‡	1083 (59.0)	6363 (39.7)
Antidepressants	547 (29.8)	3731 (23.1)
Benzodiazepines/Anxiolytics	423 (23.1)	2681 (17.2)
Antipsychotics	286 (15.6)	1694 (10.7)
Antiepileptics	198 (10.8)	1084 (7.0)
Opiates	628 (34.2)	4299 (26.9)
Barbiturates	0 (0.0)	6 (0.0)
Mood Stabilizers	14 (0.8)	134 (0.8)
Immunosuppressants	5 (0.3)	28 (0.2)
Influenza vaccination prior to index date	1529 (83.3)	13335 (82.8)

* Percentages weighted by the inverse of the number of controls per cases.

† Percentages among subjects with no missing data in these variables.

‡Excluding the 2 weeks prior to the index date.

After adjustment for differences in the covariates, current use of pramipexole was not associated with an increase in the rate of pneumonia compared with no use (rate ratio (RR) 0.76; 95% confidence interval (CI): 0.57-1.02) or when compared with current use of all other dopamine agonists collectively (RR 0.85; 95% CI: 0.62-1.17), whether ergot-derived or non-ergot-derived (Table 2). The current use of any dopamine agonist, compared with non-use, was not associated with an increase in the rate of pneumonia (RR 0.87; 95% CI, 0.75-1.02). Looking at specific agents, slightly reduced risks were found for pramipexole (RR 0.76; 95% CI, 0.57-1.02) and ropinirole (RR 0.76; 95% CI, 0.60-0.97) (Table 3).

**Table 2:** **Crude and adjusted rateratios of pneumonia associatedwith current use ofpramipexole relative to otherdopamine agonists**

	**Cases**	**Controls**	**Crude**	Adjusted*	
			**Rate Ratio**	**Rate ratio**	**95% CI**
Number of subjects	1835	16,088			
Current use† of (%)					
Pramipexole (%)	59 (3.2)	734 (4.6)	0.80	0.85	0.62 – 1.17
All other dopamine agonists (%)	210 (11.4)	2112 (13.1)	1.00	1.00	Reference
Pramipexole (%)	59 (3.2)	734 (4.6)	0.90	0.95	0.68 – 1.35
Non-Ergot-derived dopamine agonists (%)	101 (5.5)	1157 (7.2)	1.00	1.00	Reference
Pramipexole (%)	59 (3.2)	734 (4.6)	0.72	0.75	0.53 – 1.08
Ergot-derived dopamine agonists (%)	109 (5.9)	966 (6.0)	1.00	1.00	Reference

* Adjusted for current use of other anti-parkinsonian drugs, including levodopa, selegiline, rasagiline, COMT inhibitors and amantadine, and all factors listed in Table 1.

† Current use refers to a prescription ending after or within 30 days prior to the index date.

**Table 3:** **Crude and adjusted rateratios of pneumonia associatedwith current use ofthe different dopamine agonistsrelative to non-current use**

	**Cases**	**Controls**	**Crude**	Adjusted*	
			**Rate Ratio**	**Rate ratio**	**95% CI**
Number of subjects	1835	16088			
Current use† of:					
Any dopamine agonist (%)	269 (14.7)	2,846 (17.7)	0.77	0.87	0.75 – 1.02
Pramipexole (%)	59 (3.2)	734 (4.6)	0.64	0.76	0.57 – 1.02
Ropinirole (%)	92 (5.0)	1,095 (6.8)	0.69	0.76	0.60 – 0.97
Cabergoline (%)	57 (3.1)	397 (2.5)	1.15	1.28	0.94 – 1.75
Pergolide (%)	43 (2.3)	418 (2.6)	0.90	1.06	0.76 – 1.50
Other dopamine agonists‡ (%)	23 (1.3)	234 (1.5)	0.74	0.77	0.48 – 1.22

* Adjusted for one another, for current use of other anti-Parkinsonian drugs, including levodopa, selegiline, rasagiline, COMT inhibitors and amantadine, and all factors listed in Table 1.

† Current use refers to a prescription ending after or within 30 days prior to the index date.

‡ Regroups rotigotine, bromocriptine, and lisuride because of low frequencies.

Sensitivity analyses showed that the association observed with pramipexole, compared with other dopamine agonists, remains unchanged when restricting the analysis to subjects for whom the indication for treatment was Parkinson's disease (RR 0.75; 95% CI: 0.50-1.11). Moreover, the results remained similar for the sub-cohort defined with at least two prescriptions for an anti-parkinsonian drug, comparing current use of any dopamine agonist (RR 0.88; 95% CI: 0.75–1.03) and pramipexole (RR 0.75: 95% CI: 0.55-1.01) with non-use. Results were not affected substantially when changing the definition of current use from 30 days to either 0 days (RR 0.63; 95% CI: 0.45-0.88), 14 days (RR 0.71; 95% CI: 0.53-0.96), 60 days (RR 0.77; 95% CI: 0.58-1.02), or 90 days (RR 0.79; 95% CI: 0.60–1.05). To rule out channeling bias, Table 4 shows the effects of current use of pramipexole, stratified by history of pneumonia prior to cohort entry, as well as by use of dopamine agonists, ergot-derived dopamine agonists, levodopa, all in the period prior to 180 days before the index date. The effect remained similar when using the more restricted definition of pneumonia (RR 0.67; 95% CI: 0.43-1.06).

**Table 4:** **Adjusted rate ratios ofpneumonia associated with currentuse of pramipexole relativeto non-current use, stratifiedby history of pneumoniaprior to cohort entryand use of anti-parkinsonian medication prior tothe 180-day period beforethe index date**

	**Cases**	**Controls**	Adjusted*		**P-value for interaction**
			**Rate ratio**	**95% CI**	
Number of subjects	1835	16088			
History of pneumonia	4/127	23/564	0.66	0.22 – 2.00	0.81
No history of pneumonia	55/1708	711/15524	0.75	0.56 – 1.01	
Levodopa use	36/1524	461/13128	0.71	0.49 – 1.01	0.43
No levodopa use	23/311	273/2960	0.90	0.55 – 1.45	
Any dopamine agonist use	46/437	602/3993	0.67	0.48 – 0.95	0.44
No dopamine agonist use	13/1398	132/12095	0.89	0.47 – 1.66	
Ergot-derived dopamine agonist use	14/258	148/2082	0.69	0.38 – 1.24	0.80
No ergot-derived dopamine agonist use	45/1577	586/14006	0.75	0.54 – 1.04	

* Adjusted for current use of other dopamine agonists, other anti-parkinsonian drugs and selected.

factors from Table 1, namely age, duration of APK use, obesity, smoking, alcohol, cerebrovascular, ischemic heart disease, hypertension, heart failure, diabetes, edema, COPD, asthma, prior pneumonia, lung cancer, other cancer, depression, anemia, renal failure, vaccination (influenza and pneumonia), PPIs, NSAIDs, oral corticosteroids, other respiratory medications, antibiotics, antipsychotics and antidepressants.

‡ Current use refers to a prescription ending after or within 30 days prior to the index date.

‡ Previous timing of covariate refers to the period prior to 180 days before the index date.

## Discussion

Using a large population-based cohort of users of anti-Parkinsonian drugs, we found that the use of pramipexole is not associated with an increase in the risk of severe pneumonia requiring hospitalization.

Community-acquired pneumonia is common in the elderly, with an estimated incidence of 15.4 per 1000 persons per year at ages 60–74 years and 34.2 at age greater than 75 years [14]. Parkinson's disease has been associated with a significant increase in deaths from pneumonia and in particular aspiration pneumonia [3-5]. Swallowing impairment is a frequent finding in Parkinson's disease, occurring in about 50% of patients [15]. Aspiration pneumonia may develop due to the infiltration of foreign materials into the bronchial tree, usually oral or gastric contents. The effect of dopaminergic treatment on swallowing in Parkinson's disease has been little studied and no consistent effect found [16,17]. It is possible that the slightly reduced risk of pneumonia observed with current therapy with pramipexole and ropinirole might be related to a decrease in aspiration into the bronchial tree. Another explanation is the presence of residual confounding, although we adjusted for treatment indication, disease duration and use of other anti-parkinsonian medications.

This study has strengths and limitations. We assembled a large population-based cohort of over 13,000 patients observed over 12 years, a size and follow-up that enabled the identification of a large number of incident cases of pneumonia and precise estimates of the risk. Selection bias was avoided by the completeness of the population-based cohort, which also provides generalizability. The possibility of recall bias in terms of exposure to dopamine agonists is avoided because the GPRD uses pre-recorded medication exposure histories. While we adjusted the risk estimates for several major confounders, there may have been some residual confounding from unmeasured covariates such as disease severity, as we did not have information on the severity of motor and non-motor symptoms of the disease or of the presence of motor complications of medications, for example. We did adjust for the duration of the disease using the first prescription of dopaminergic medication as an indirect proxy for disease severity. Also, the concomitant use of other dopaminergic treatments, indicating a more advanced disease was taken into account. It is unlikely that such confounding had much effect on the comparative risks among dopamine agonists, as there is no evidence that the choice of agent is related to severity. Despite its size, our study was not sufficiently large for the stratification needed to study the effects of dose and duration of pramipexole use.

## Conclusion

In summary, the use of pramipexole and of other dopamine agonists, did not appear to increase the risk of pneumonia in this study population.

## Competing interests

Dr. Ernst has received speaker fees and has attended advisory boards for AstraZeneca, Boehringer Ingelheim, GlaxoSmithKline, Merck, Novartis, and Nycomed. Dr. Suissa has received research grants from AstraZeneca, Boehringer Ingelheim and GlaxoSmithKline, and has participated in advisory board meetings and as speaker in conferences for AstraZeneca, Boehringer-Ingelheim, GlaxoSmithKline, Novartis, Pfizer and Merck.

## Authors' contributions

All authors took part in designing the study. SS and SD carried out the statistical analysis. PE and CR drafted the manuscript. All authors read and approved the final manuscript.

## Pre-publication history

The pre-publication history for this paper can be accessed here:

http://www.biomedcentral.com/1471-2377/12/113/prepub
